# On estimation procedures of stress-strength reliability for Weibull distribution with application

**DOI:** 10.1371/journal.pone.0237997

**Published:** 2020-08-24

**Authors:** Abdullah M. Almarashi, Ali Algarni, Mazen Nassar

**Affiliations:** Department of Statistics, Faculty of Science, King Abdulaziz University, Jeddah, Kingdom of Saudia Arabia; Tongii University, CHINA

## Abstract

For the first time, ten frequentist estimation methods are considered on stress-strength reliability *R* = *P*(*Y* < *X*) when *X* and *Y* are two independent Weibull distributions with the same shape parameter. The start point to estimate the parameter *R* is the maximum likelihood method. Other than the maximum likelihood method, a nine frequentist estimation methods are used to estimate *R*, namely: least square, weighted least square, percentile, maximum product of spacing, minimum spacing absolute distance, minimum spacing absolute-log distance, method of Cramér-von Mises, Anderson-Darling and Right-tail Anderson-Darling. We also consider two parametric bootstrap confidence intervals of *R*. We compare the efficiency of the different proposed estimators by conducting an extensive Mont Carlo simulation study. The performance and the finite sample properties of the different estimators are compared in terms of relative biases and relative mean squared errors. The Mont Carlo simulation study revels that the percentile and maximum product of spacing methods are highly competitive with the other methods for small and large sample sizes. To show the applicability and the importance of the proposed estimators, we analyze one real data set.

## 1 Introduction

Since the pioneering work of Birnbaum [1], the statistical inference of the stress-strength parameter have received considerable attention and commonly used in many engineering applications. Let *X* and *Y* be two independent random variables showing the strength of a system and the stress applied to it, respectively, then *R* = *P*(*Y* < *X*) is the measure of the system performance which arises quite naturally in the mechanical reliability of a system. In this case the system fails if and only if the applied stress is larger than its strength at any time. In the statistical literature, several authors considered the estimation problem of the stress strength parameter. [2, 3] studied the estimation of *R*, where *X* and *Y* are generalized exponential and Weibull random variables, respectively. [4] obtained the estimation of *R*, where *X* and *Y* are distributed as two independent three parameter generalized exponential random variables. [5] considered the estimation of *R*, where *X* and *Y* are two independent generalized Pareto distributions. In recent years, a many works have been done on the problem of estimating *R* of some distributions; See for example, [6–14] and [15]. These studies have actually considered the maximum likelihood and Bayesian estimation methods under different life testing schemes for various distributions.

Many authors investigated the estimation of *R* = *P*(*Y* < *X*) when *X* and *Y* are independent Weibull random variables, see for example [3, 7, 16–18], and see the references cited therein. These studies considered the maximum likelihood as a frequentist method as well as the Bayesian estimation method. But no much attention has been paid to use other frequentist methods to estimate *R* = *P*(*Y* < *X*), while in some cases they can provide better estimates than the maximum likelihood method. For this reason, our aim in this paper is to use nine frequentist methods of estimation besides the maximum likelihood estimation (MLE) method. In this paper, we focus on ten different estimation methods to estimate the stress-strength reliability and investigate how the different estimators behave for different sample sizes and for different parameter values. Further, we develop a guideline for choosing the best estimation method to estimate the stress-strength reliability of the Weibull distribution, which we think would be of deep interest to applied statisticians/reliability engineers. In addition, the simulation study and the real data analysis prove that there are classical methods rather than the MLE methods which can be give a desirable estimates, motivating its use in applied areas. Also, it is to be mentioned that it is the first time to consider ten estimation methods of estimation to estimate the stress-strength reliability of the Weibull distribution. These methods are: least square estimation (LSE), weighted least square estimation (WLSE), percentile estimation (PCE), maximum product of spacing estimation (MPSE), minimum spacing absolute distance estimation (MSADE), minimum spacing absolute-log distance estimation (MSALDE), method of Cramér-von-Mises estimation (CME), Anderson-Darling estimation (ADE) and Right-tail Anderson-Darling estimation (RTADE) methods. For more details about these estimation methods one can refer to [19–21] and [22]. We are aware of no studies used these frequentist methods of estimation to estimate *R* = *P*(*Y* < *X*) when *X* and *Y* are independent Weibull random variables. Therefore, it is the first time to use the aforementioned ten estimation methods to estimate the stress-strength parameter. The Weibull distribution with scale parameter *β* and shape parameter *α*, denoted by *W*(*β*, *α*) has the following probability density function (PDF)

$$\begin{array}{c} f\left(x;\beta ,\alpha \right)=\beta \alpha x^{\alpha -1}e^{-\beta x^{\alpha }},\;>0,\beta ,\alpha >0 \end{array}$$ (1)

and the corresponding cumulative distribution function (CDF) is given by

$$\begin{array}{c} F\left(x;\beta ,\alpha \right)=1-e^{-\beta x^{\alpha }}. \end{array}$$ (2)

Let *X* and *Y* are independent Weibull random variables and follow *W*(*β*_1_, *α*) and *W*(*β*_2_, *α*), respectively, then *R* = *P*(*Y* < *X*) can be written as follows, see [23],

$$\begin{array}{c} R=\frac{\beta_{1}}{\beta_{1}+\beta_{2}} \end{array}$$ (3)

The main objective of this study is to use the aforementioned ten estimation methods to estimate the stress-strength reliability when *X* and *Y* are independent Weibull random variables with the same shape parameter. Furthermore, we use two parametric bootstrap confidence intervals of the stress-strength parameter. We perform an extensive Mont Carlo simulation study to compare the efficiency of the different estimates as well as a real data set is analyzed for illustrative purpose. The rest of this paper is organized as follows: In Section 2, we discuss the different estimation methods. In Section 3, different bootstrap confidence intervals are presented. A Mont Carlo simulation study is performed in Section 4. A real data set is analyzed in Section 5. Finally, the paper is concluded in Section 6.

## 2 Different estimation methods

In this section, we describe the ten frequentist methods of estimation considered in this paper to obtain the different estimates of the stress-strength parameter. In the literature there are several classical methods that can be used to estimate the stress-strength parameter rather than the MLE, where the MLE does not perform well in many cases especially for small samples. Therefore, considering other frequentist estimation methods will be of main interest to compare such methods with other the MLE procedure.

### 2.1 Maximum likelihood estimation

Let *x*_1_, …, *x*_*m*_ and *y*_1_, …, *y*_*r*_ be a random samples from *W*(*α*, *β*_1_) and *W*(*α*, *β*_2_), respectively, then we can write the natural logarithm of the likelihood function of the observed sample as follows

$$\begin{array}{ccc} L(\beta_{1},\beta_{2},\alpha ) & = & \left(m+r\right)\;\text{log}\;\left(\alpha \right)+m\;\text{log}\;\left(\beta_{1}\right)+r\;\text{log}\;\left(\beta_{2}\right)\\ & + & \left(\alpha -1\right)[\sum_{i=1}^{m}\;\text{log}\;\left(x_{i}\right)+\sum_{j=1}^{r}\;\text{log}\;\left(y_{j}\right)]-\beta_{1}\sum_{i=1}^{m}x_{i}^{\alpha }-\beta_{2}\sum_{j=1}^{r}y_{j}^{\alpha }. \end{array}$$ (4)

The maximum likelihood estimators (MLEs) of *β*_1_, *β*_2_ and *α* denoted by $\beta_{1}^{MLE},\beta_{2}^{MLE}$ and *α*^*MLE*^, respectively, can be obtained by solving the following equations

$$\begin{array}{c} \frac{\partial L(\beta_{1},\beta_{2},\alpha )}{\partial \beta_{1}}=\frac{m}{\beta_{1}}-\sum_{i=1}^{m}x_{i}^{\alpha }, \end{array}$$ (5)

$$\begin{array}{c} \frac{\partial L(\beta_{1},\beta_{2},\alpha )}{\partial \beta_{2}}=\frac{r}{\beta_{2}}-\sum_{j=1}^{r}y_{j}^{\alpha } \end{array}$$ (6)

and

$$\begin{array}{c} \frac{\partial L(\beta_{1},\beta_{2},\alpha )}{\partial \alpha }=\frac{m+r}{\beta }+\sum_{i=1}^{m}\;\text{log}\;\left(x_{i}\right)+\sum_{j=1}^{r}\;\text{log}\;\left(y_{j}\right)-\beta_{1}\sum_{i=1}^{m}x_{i}^{\alpha }\;\text{log}\;\left(x_{i}\right)-\beta_{2}\sum_{j=1}^{r}y_{j}^{\alpha }\;\text{log}\;\left(y_{j}\right). \end{array}$$ (7)

The $\beta_{1}^{MLE}$ and $\beta_{2}^{MLE}$ can be obtained from (5) and (6), respectively, as a function of the unknown parameter *α* as follow

$$\begin{array}{c} \beta_{1}^{MLE}\left(\alpha \right)=\frac{m}{\sum_{i=1}^{m}y_{i}^{\alpha }}\;\;\;\;\;\;\text{and}\;\;\;\;\;\;\beta_{2}^{MLE}\left(\alpha \right)=\frac{r}{\sum_{j=1}^{r}z_{j}^{\alpha }}. \end{array}$$ (8)

Substituting the estimators $\beta_{1}^{MLE}\left(\alpha \right)$ and $\beta_{2}^{MLE}\left(\alpha \right)$ obtained from (8) in (4), then we can obtain the profile log-likelihood function of the parameter *α* as follows

$$\begin{array}{c} L\left(\alpha \right)=\left(m+r\right)\;\text{log}\;\left(\alpha \right)+\alpha [\sum_{i=1}^{m}\;\text{log}\;\left(x_{i}\right)+\sum_{j=1}^{r}\;\text{log}\;\left(y_{j}\right)]-m\;\text{log}\;(\sum_{i=1}^{m}x_{i}^{\alpha })-r\;\text{log}\;(\sum_{j=1}^{r}y_{j}^{\alpha }). \end{array}$$ (9)

To obtain *α*^*MLE*^, we differentiate (9) with respect to *α* and equating result by zero, therefore

$$\begin{array}{c} \psi \left(\alpha \right)={\left(\frac{m\sum_{i=1}^{m}x_{i}^{\alpha }\;\text{log}\;\left(x_{i}\right)}{\left(m+r\right)\sum_{i=1}^{m}x_{i}^{\alpha }}+\frac{r\sum_{j=1}^{m}y_{j}^{\alpha }\;\text{log}\;\left(y_{j}\right)}{\left(m+r\right)\sum_{j=1}^{m}y_{j}^{\alpha }}-\frac{\sum_{i=1}^{m}\;\text{log}\;\left(x_{i}\right)+\sum_{j=1}^{r}\;\text{log}\;\left(y_{j}\right)}{m+r}\right)}^{-1}. \end{array}$$ (10)

After obtaining the *α*^*MLE*^ from (10) by using any iteration procedure, we can obtain $\beta_{1}^{MLE}$ and $\beta_{2}^{MLE}$ from (8), for more details about the method of maximum likelihood see for example [24–26]. Now, the MLE of *R* can be obtained as follows

$$\begin{array}{c} {\hat{R}}^{MLE}=\frac{\beta_{1}^{MLE}}{\beta_{1}^{MLE}+\beta_{2}^{MLE}}. \end{array}$$

### 2.2 Least square and weighted least square estimation

The least square and weighted least square methods of estimation were suggested by [27] to estimate the parameters of Beta distribution. Let *x*_1:*m*_, …, *x*_*m*:*m*_ be the order statistics of a random sample of size *m* from *W*(*α*, *β*_1_) and *y*_1:*r*_, …, *y*_*r*:*r*_ be the order statistics of a random sample of size *r* from *W*(*α*, *β*_2_). The LSEs of the unknown parameters *β*_1_, *β*_2_ and *α* denoted by ${\hat{\beta_{1}}}^{LSE},{\hat{\beta_{2}}}^{LSE}$ and ${\hat{\alpha }}^{LSE}$ can be obtained by minimizing the following function with respect to *β*_1_, *β*_2_ and *α*

$$\begin{array}{ccc} Ls(\beta_{1},\beta_{2},\alpha ) & = & \sum_{i=1}^{m}{\left[F\left(x_{i:m}\right)-\frac{i}{m+1}\right]}^{2}+\sum_{j=1}^{r}{\left[F\left(y_{j:r}\right)-\frac{j}{r+1}\right]}^{2}\\ & = & \sum_{i=1}^{m}{\left[\frac{m+1-i}{m+1}-e^{-\beta_{1}x_{i:m}^{\alpha }}\right]}^{2}+\sum_{j=1}^{r}{\left[\frac{r+1-j}{r+1}-e^{-\beta_{2}y_{j:r}^{\alpha }}\right]}^{2}. \end{array}$$ (11)

Instead of minimizing (11), the estimates ${\hat{\beta_{1}}}^{LSE},{\hat{\beta_{2}}}^{LSE}$ and ${\hat{\alpha }}^{LSE}$ can be obtained by solving the following three equations simultaneously

$$\begin{array}{c} \frac{\partial Ls}{\partial \beta_{1}}=\sum_{i=1}^{m}y_{i:m}^{\alpha }e^{-\beta_{1}y_{i:m}^{\alpha }}[\frac{m+1-i}{m+1}-e^{-\beta_{1}x_{i:m}^{\alpha }}]=0,\\ \frac{\partial Ls}{\partial \beta_{2}}=\sum_{j=1}^{r}y_{j:r}^{\alpha }e^{-\beta_{2}y_{j:r}^{\alpha }}[\frac{r+1-j}{r+1}-e^{-\beta_{2}y_{j:r}^{\alpha }}]=0 \end{array}$$

and

$$\begin{array}{ccc} \frac{\partial Ls}{\partial \alpha } & = & \beta_{1}\sum_{i=1}^{m}x_{i:m}^{\alpha }\;\text{log}\;\left(x_{i:m}\right)e^{-\beta_{1}x_{i:m}^{\alpha }}[\frac{m+1-i}{m+1}-e^{-\beta_{1}x_{i:m}^{\alpha }}]\\ & + & \beta_{2}\sum_{j=1}^{r}y_{j:r}^{\alpha }\;\text{log}\;\left(y_{j:r}\right)e^{-\beta_{1}y_{j:r}^{\alpha }}[\frac{r+1-j}{r+1}-e^{-\beta_{2}y_{j:r}^{\alpha }}]=0, \end{array}$$

where *Ls* ≡ *Ls*(*β*_1_, *β*_2_, *α*). Upon obtaining the estimates ${\hat{\beta_{1}}}^{LSE},{\hat{\beta_{2}}}^{LSE}$ and ${\hat{\alpha }}^{LSE}$, the LSE of *R* can be obtained as follows

$$\begin{array}{c} {\hat{R}}^{LSE}=\frac{\beta_{1}^{LSE}}{\beta_{1}^{LSE}+\beta_{2}^{LSE}}. \end{array}$$

Similarly, the WLSEs of the the unknown parameters *β*_1_, *β*_2_ and *α* denoted by ${\hat{\beta_{1}}}^{WLSE},{\hat{\beta_{2}}}^{WLSE}$ and ${\hat{\alpha }}^{WLSE}$ can be obtained by minimizing the following function

$$\begin{array}{ccc} Ws & = & \sum_{i=1}^{m}\omega_{1}\left(i,m\right){\left[\frac{m+1-i}{m+1}-e^{-\beta_{1}x_{i:m}^{\alpha }}\right]}^{2}+\sum_{j=1}^{r}\omega_{2}\left(j,r\right){\left[\frac{r+1-j}{r+1}-e^{-\beta_{2}y_{j:r}^{\alpha }}\right]}^{2}, \end{array}$$ (12)

with respect to *β*_1_, *β*_2_ and *α*, where $\omega_{1}\left(i,m\right)=\frac{{\left(m+1\right)}^{2}\left(m+2\right)}{i(m-i+1)}$ and $\omega_{2}\left(j,r\right)=\frac{{\left(r+1\right)}^{2}\left(r+2\right)}{j(r-j+1)}$, where *Ws* ≡ *Ws*(*β*_1_, *β*_2_, *α*). These estimates can also be obtained by solving the following three equations simultaneously

$$\begin{array}{c} \frac{\partial Ws}{\partial \beta_{1}}=\sum_{i=1}^{m}\omega_{1}\left(i,m\right)x_{i:m}^{\alpha }e^{-\beta_{1}x_{i:m}^{\alpha }}[\frac{m+1-i}{m+1}-e^{-\beta_{1}x_{i:m}^{\alpha }}]=0,\\ \frac{\partial Ws}{\partial \beta_{2}}=\sum_{i=1}^{r}\omega_{2}\left(i,r\right)y_{j:r}^{\alpha }e^{-\beta_{2}y_{j:r}^{\alpha }}[\frac{r+1-j}{r+1}-e^{-\beta_{2}y_{j:r}^{\alpha }}]=0 \end{array}$$

and

$$\begin{array}{ccc} \frac{\partial Ws}{\partial \alpha } & = & \beta_{1}\sum_{i=1}^{m}\omega_{1}\left(i,m\right)x_{i:m}^{\alpha }\;\text{log}\;\left(x_{i:m}\right)e^{-\beta_{1}x_{i:m}^{\alpha }}[\frac{m+1-i}{m+1}-e^{-\beta_{1}x_{i:m}^{\alpha }}]\\ & + & \beta_{2}\sum_{j=1}^{r}\omega_{2}\left(j,r\right)y_{j:r}^{\alpha }\;\text{log}\;\left(y_{j:r}\right)e^{-\beta_{1}y_{j:r}^{\alpha }}[\frac{r+1-j}{r+1}-e^{-\beta_{2}y_{j:r}^{\alpha }}]=0. \end{array}$$

The WLSE of *R* can be obtained as follows

$$\begin{array}{c} {\hat{R}}^{WLSE}=\frac{\beta_{1}^{WLSE}}{\beta_{1}^{WLSE}+\beta_{2}^{WLSE}}. \end{array}$$

### 2.3 Percentile estimation

The percentile method of estimation is introduced by [28, 29] and recently used by many authors, see for example [30] and [31]. Since the Weibull distribution has an explicit CDF as shown in (2), therefore we can obtain the estimates of the unknown parameters *β*_1_, *β*_2_ and *α* by equating the sample percentile points with the population percentile points. Let $p_{1i}=\frac{i}{m+1}$ is the unbiased estimator of *F*(*y*_*i*:*m*_|*β*_1_, *α*) and $p_{2j}=\frac{j}{r+1}$ is the unbiased estimator of *F*(*z*_*i*:*r*_|*β*_2_, *α*), then the PCEs estimators denoted by ${\hat{\beta_{1}}}^{PCE},{\hat{\beta_{2}}}^{PCE}$ and ${\hat{\alpha }}^{PCE}$ can be obtained by minimizing, with respect to *β*_1_, *β*_2_ and *α*, the following function

$$\begin{array}{ccc} Pe & = & \sum_{i=1}^{m}{\left[x_{i:m}-{\left(-\frac{\;\text{log}\;(1-p_{1i})}{\beta_{1}}\right)}^{1/\alpha }\right]}^{2}+\sum_{j=1}^{r}{\left[y_{j:r}-{\left(-\frac{\;\text{log}\;(1-p_{2j})}{\beta_{2}}\right)}^{1/\alpha }\right]}^{2}, \end{array}$$ (13)

where *Pe* ≡ *Pe*(*β*_1_, *β*_2_, *α*). The estimates obtained from (13) can be also obtained by solving the following three equations simultaneously

$$\begin{array}{l} \frac{\partial Pe}{\partial \beta_{1}}=\frac{1}{\alpha \beta_{1}^{2}}\sum_{i=1}^{m}[x_{i:m}-{\left(-\frac{\;\text{log}\;(p_{1i}^{*})}{\beta_{1}}\right)}^{1/\alpha }]{\left(\frac{\;\text{log}\;(p_{1i}^{*})}{\beta_{1}}\right)}^{1/\alpha -1}\;\text{log}\;(p_{1i}^{*})=0,\\ \frac{\partial Pe}{\partial \beta_{2}}=\frac{1}{\alpha \beta_{2}^{2}}\sum_{j=1}^{r}[y_{j:r}-{\left(-\frac{\;\text{log}\;(p_{2i}^{*})}{\beta_{2}}\right)}^{1/\alpha }]{\left(\frac{\;\text{log}\;(p_{2i}^{*})}{\beta_{2}}\right)}^{1/\alpha -1}\;\text{log}\;(p_{2i}^{*})=0, \end{array}$$

and

$$\begin{array}{ccc} \frac{\partial Pe}{\partial \alpha } & = & \frac{1}{\alpha^{2}}\sum_{i=1}^{m}[x_{i:m}-{\left(-\frac{\;\text{log}\;(p_{1i}^{*})}{\beta_{1}}\right)}^{1/\alpha }]{\left(-\frac{\;\text{log}\;(p_{1i}^{*})}{\beta_{1}}\right)}^{1/\alpha }\;\text{log}\;(-\frac{\;\text{log}\;(p_{1i}^{*})}{\beta_{1}})\\ & + & \frac{1}{\alpha^{2}}\sum_{j=1}^{r}[y_{j:r}-{\left(-\frac{\;\text{log}\;(p_{2i}^{*})}{\beta_{2}}\right)}^{1/\alpha }]{\left(-\frac{\;\text{log}\;(p_{2i}^{*})}{\beta_{2}}\right)}^{1/\alpha }\;\text{log}\;(-\frac{\;\text{log}\;(p_{2i}^{*})}{\beta_{2}})=0, \end{array}$$

where $p_{1i}^{*}=1-p_{1i}$ and $p_{2i}^{*}=1-p_{2i}$. Based on the estimates ${\hat{\beta_{1}}}^{PCE},{\hat{\beta_{2}}}^{PCE}$ and ${\hat{\alpha }}^{PCE}$, the PCE of *R* can be obtained as follows

$$\begin{array}{c} {\hat{R}}^{PCE}=\frac{\beta_{1}^{PCE}}{\beta_{1}^{PCE}+\beta_{2}^{PCE}}. \end{array}$$

### 2.4 Maximum product of estimation

The method of maximum product of spacing is proposed by [32, 33] as an alternative to the maximum likelihood method to estimate the parameters of continuous univariate distribu1tions. Let *y*_1:*m*_, …, *y*_*m*:*m*_ be the order statistics of a random sample of size *m* from *W*(*α*, *β*_1_) and *z*_1:*r*_, …, *z*_*r*:*r*_ be the order statistics of a random sample of size *r* from *W*(*α*, *β*_2_), then we can define the uniform spacings of the two samples, respectively, as

$$\begin{array}{c} {△}_{1i}=F\left(x_{i:m}|\beta_{1},\alpha \right)-F\left(x_{i-1:m}|\beta_{1},\alpha \right) \end{array}$$

and

$$\begin{array}{c} {△}_{2j}=F\left(y_{j:r}|\beta_{2},\alpha \right)-F\left(y_{j-1:r}|\beta_{2},\alpha \right). \end{array}$$

Using the same approach as in Cheng and Amin (1983), the MPSEs of the unknown parameters are obtained by maximizing the following function

$$\begin{array}{ccc} MP(\beta_{1},\beta_{2},\alpha ) & = & \frac{1}{m+1}\sum_{i=1}^{m+1}\;\text{log}\;({△}_{1i})+\frac{1}{r+1}\sum_{j=1}^{r+1}\;\text{log}\;({△}_{2j}). \end{array}$$ (14)

From (2) and (14), the MPSEs of the unknown parameters *β*_1_, *β*_2_ and *α* denoted by ${\hat{\beta_{1}}}^{MPSE},{\hat{\beta_{2}}}^{MPSE}$ and ${\hat{\alpha }}^{MPSE}$ can be obtained by maximizing, with respect to *β*_1_, *β*_2_ and *α*, the following function

$$\begin{array}{ccc} MP & = & \frac{1}{m+1}\sum_{i=1}^{m+1}\;\text{log}\;(e^{-\beta_{1}x_{i-1:m}^{\alpha }}-e^{-\beta_{1}x_{i:m}^{\alpha }})+\frac{1}{r+1}\sum_{j=1}^{r+1}\;\text{log}\;(e^{-\beta_{2}y_{j-1:r}^{\alpha }}-e^{-\beta_{2}y_{j:r}^{\alpha }}), \end{array}$$

where *MP* ≡ *MP*(*β*_1_, *β*_2_, *α*). Equivalently these estimates can be obtained by solving the following three equations simultaneously

$$\begin{array}{c} \frac{\partial MP}{\partial \beta_{1}}=\frac{1}{m+1}\sum_{i=1}^{m+1}\frac{x_{i-1:m}^{\alpha }e^{-\beta_{1}x_{i-1:m}^{\alpha }}-x_{i:m}^{\alpha }e^{-\beta_{1}x_{i:m}^{\alpha }}}{e^{-\beta_{1}x_{i-1:m}^{\alpha }}-e^{-\beta_{1}x_{i:m}^{\alpha }}}=0,\\ \frac{\partial MP)}{\partial \beta_{2}}=\frac{1}{r+1}\sum_{j=1}^{r+1}\frac{y_{j-1:r}^{\alpha }e^{-\beta_{2}y_{j-1:r}^{\alpha }}-y_{j:r}^{\alpha }e^{-\beta_{2}y_{j:r}^{\alpha }}}{e^{-\beta_{2}y_{j-1:r}^{\alpha }}-e^{-\beta_{2}y_{j:r}^{\alpha }}}=0 \end{array}$$

and

$$\begin{array}{c} \frac{\partial MP}{\partial \alpha }=\frac{\beta_{1}}{m+1}\sum_{i=1}^{m+1}\frac{w_{i-1:m}-w_{i:m}}{e^{-\beta_{1}x_{i-1:m}^{\alpha }}-e^{-\beta_{1}x_{i:m}^{\alpha }}}+\frac{\beta_{2}}{r+1}\sum_{j=1}^{r+1}\frac{t_{j-1:r}-t_{j:r}}{e^{-\beta_{2}y_{j-1:r}^{\alpha }}-e^{-\beta_{2}y_{j:r}^{\alpha }}}=0, \end{array}$$

where $w_{i:m}=x_{i:m}^{\alpha }e^{-\beta_{1}x_{i:m}^{\alpha }}\;\text{log}\;\left(y_{i:m}\right)$ and $t_{j:r}=z_{j:r}^{\alpha }e^{-\beta_{2}z_{j:r}^{\alpha }}\;\text{log}\;\left(z_{j:r}\right)$. Using the obtained estimates, we can obtain the MPSE of *R* as follows

$$\begin{array}{c} {\hat{R}}^{MPSE}=\frac{\beta_{1}^{MPSE}}{\beta_{1}^{MPSE}+\beta_{2}^{MPSE}}. \end{array}$$

### 2.5 Minimum spacing distance estimation

The method of minimum spacing distance estimation was originally proposed by [34]. Using the same notations in the previous subsections, the minimum spacing distance estimators are obtained by minimizing the following function

$$\begin{array}{ccc} MD(\beta_{1},\beta_{2},\alpha ) & = & \sum_{i=1}^{m+1}\psi ({△}_{1i},\phi_{1}\left(m\right))+\sum_{j=1}^{r+1}\psi ({△}_{2j},\phi_{2}\left(r\right)), \end{array}$$ (15)

where $\phi_{1}\left(m\right)=\frac{1}{m+1}$, $\phi_{2}\left(r\right)=\frac{1}{r+1}$ and *ψ*(*a*, *b*) is an appropriate distance. The common choices of *ψ*(*a*, *b*) in (15) are |*a* − *b*| and |log(*a*) − log(*b*)|, which are called absolute and absolute-log distance, respectively. The MSADEs of the unknown parameters denoted by ${\hat{\beta_{1}}}^{MSADE},{\hat{\beta_{2}}}^{MSADE}$ and ${\hat{\alpha }}^{MSADE}$ can be obtained by minimizing the following function with respect to *β*_1_, *β*_2_ and *α*

$$\begin{array}{ccc} Ma & = & \sum_{i=1}^{m+1}|e^{-\beta_{1}x_{i-1:m}^{\alpha }}-e^{-\beta_{1}x_{i:m}^{\alpha }}-\phi_{1}\left(m\right)|+\sum_{j=1}^{r+1}\left|e^{-\beta_{2}y_{j-1:r}^{\alpha }}-e^{-\beta_{2}y_{j:r}^{\alpha }}-\phi_{2}\left(r\right)\right|, \end{array}$$ (16)

where *Ma* ≡ *MA*(*β*_1_, *β*_2_, *α*), or by solving the following three equations simultaneously

$$\begin{array}{c} \frac{\partial Ma}{\partial \beta_{1}}=\sum_{i=1}^{m+1}\frac{e^{-\beta_{1}x_{i-1:m}^{\alpha }}-e^{-\beta_{1}x_{i:m}^{\alpha }}-\phi_{1}\left(m\right)}{|e^{-\beta_{1}x_{i-1:m}^{\alpha }}-e^{-\beta_{1}x_{i:m}^{\alpha }}-\phi_{1}\left(m\right)|}(x_{i-1:m}^{\alpha }e^{-\beta_{1}x_{i-1:m}^{\alpha }}-x_{i:m}^{\alpha }e^{-\beta_{1}x_{i:m}^{\alpha }})=0,\\ \frac{\partial Ma}{\partial \beta_{2}}=\sum_{j=1}^{r+1}\frac{e^{-\beta_{2}y_{j-1:r}^{\alpha }}-e^{-\beta_{2}y_{j:r}^{\alpha }}-\phi_{2}\left(r\right)}{|e^{-\beta_{2}y_{j-1:r}^{\alpha }}-e^{-\beta_{2}y_{j:r}^{\alpha }}-\phi_{2}\left(r\right)|}(y_{j-1:r}^{\alpha }e^{-\beta_{2}y_{j-1:r}^{\alpha }}-z_{j:r}^{\alpha }e^{-\beta_{2}y_{j:r}^{\alpha }})=0 \end{array}$$

and

$$\begin{array}{ccc} \frac{\partial Ma}{\partial \alpha } & = & \beta_{1}\sum_{i=1}^{m+1}\frac{\left(e^{-\beta_{1}x_{i-1:m}^{\alpha }}-e^{-\beta_{1}x_{i:m}^{\alpha }}-\phi_{1}\left(m\right))(w_{i-1:m}-w_{i:m}\right)}{|e^{-\beta_{1}x_{i-1:m}^{\alpha }}-e^{-\beta_{1}x_{i:m}^{\alpha }}-\phi_{1}\left(m\right)|}\\ & + & \beta_{2}\sum_{j=1}^{r+1}\frac{\left(e^{-\beta_{2}y_{j-1:r}^{\alpha }}-e^{-\beta_{2}y_{j:r}^{\alpha }}-\phi_{2}\left(r\right))(t_{j-1:r}-t_{j:r}\right)}{|e^{-\beta_{2}y_{j-1:r}^{\alpha }}-e^{-\beta_{2}y_{j:r}^{\alpha }}-\phi_{2}\left(r\right)|}=0. \end{array}$$

Similarly, the MSALDEs of the the unknown parameters *β*_1_, *β*_2_ and *α* denoted by ${\hat{\beta_{1}}}^{MSALDE},{\hat{\beta_{2}}}^{MSALDE}$ and ${\hat{\alpha }}^{MSALDE}$ can be obtained by minimizing the following function

$$\begin{array}{ccc} Ml & = & \sum_{i=1}^{m+1}|\;\text{log}\;(\frac{e^{-\beta_{1}x_{i-1:m}^{\alpha }}-e^{-\beta_{1}x_{i:m}^{\alpha }}}{\phi_{1}\left(m\right)})|+\sum_{j=1}^{r+1}|\;\text{log}\;(\frac{e^{-\beta_{2}y_{j-1:r}^{\alpha }}-e^{-\beta_{2}y_{j:r}^{\alpha }}}{\phi_{2}\left(r\right)})|, \end{array}$$

where *Ml* ≡ *Ml*(*β*_1_, *β*_2_, *α*), or by solving the following three equations simultaneously

$$\begin{array}{ccc} \frac{\partial Ml}{\partial \beta_{1}} & = & \sum_{i=1}^{m+1}\frac{\left[\;\text{log}\;(e^{-\beta_{1}x_{i-1:m}^{\alpha }}-e^{-\beta_{1}x_{i:m}^{\alpha }})-\;\text{log}\;\left(\phi_{1}\left(m\right)\right)\right]}{|\;\text{log}\;(e^{-\beta_{1}x_{i-1:m}^{\alpha }}-e^{-\beta_{1}x_{i:m}^{\alpha }})-\;\text{log}\;\left(\phi_{1}\left(m\right)\right)|(e^{-\beta_{1}x_{i-1:m}^{\alpha }}-e^{-\beta_{1}x_{i:m}^{\alpha }})}\\ & \times & (x_{i-1:m}^{\alpha }e^{-\beta_{1}x_{i-1:m}^{\alpha }}-x_{i:m}^{\alpha }e^{-\beta_{1}x_{i:m}^{\alpha }})=0,\\ \frac{\partial Ml}{\partial \beta_{2}} & = & \sum_{j=1}^{r+1}\frac{\left[\;\text{log}\;(e^{-\beta_{1}y_{j-1:r}^{\alpha }}-e^{-\beta_{2}y_{j:r}^{\alpha }})-\;\text{log}\;\left(\phi_{2}\left(r\right)\right)\right]}{|\;\text{log}\;(e^{-\beta_{2}y_{j-1:r}^{\alpha }}-e^{-\beta_{2}y_{j:r}^{\alpha }})-\;\text{log}\;\left(\phi_{2}\left(r\right)\right)|(e^{-\beta_{2}y_{j-1:r}^{\alpha }}-e^{-\beta_{2}y_{j:r}^{\alpha }})}\\ & \times & (y_{j-1:r}^{\alpha }e^{-\beta_{2}y_{j-1:r}^{\alpha }}-y_{j:r}^{\alpha }e^{-\beta_{2}y_{j:r}^{\alpha }})=0 \end{array}$$

and

$$\begin{array}{ccc} \frac{\partial Ml}{\partial \alpha } & = & \beta_{1}\sum_{i=1}^{m+1}\frac{\left[\;\text{log}\;(e^{-\beta_{1}x_{i-1:m}^{\alpha }}-e^{-\beta_{1}x_{i:m}^{\alpha }})-\;\text{log}\;\left(\phi_{1}\left(m\right)\right)](w_{i-1:m}-w_{i:m}\right)}{|\;\text{log}\;(e^{-\beta_{1}x_{i-1:m}^{\alpha }}-e^{-\beta_{1}x_{i:m}^{\alpha }})-\;\text{log}\;\left(\phi_{1}\left(m\right)\right)|(e^{-\beta_{1}x_{i-1:m}^{\alpha }}-e^{-\beta_{1}x_{i:m}^{\alpha }})}\\ & + & \beta_{2}\sum_{j=1}^{r+1}\frac{\left[\;\text{log}\;(e^{-\beta_{1}y_{j-1:r}^{\alpha }}-e^{-\beta_{2}y_{j:r}^{\alpha }})-\;\text{log}\;\left(\phi_{2}\left(r\right)\right)](t_{j-1:r}-t_{j:r}\right)}{|\;\text{log}\;(e^{-\beta_{2}y_{j-1:r}^{\alpha }}-e^{-\beta_{2}y_{j:r}^{\alpha }})-\;\text{log}\;\left(\phi_{2}\left(r\right)\right)|(e^{-\beta_{2}y_{j-1:r}^{\alpha }}-e^{-\beta_{2}y_{j:r}^{\alpha }})}=0. \end{array}$$

Now, the MSADE and MSALDE of *R* can be obtained, respectively, by

$$\begin{array}{c} {\hat{R}}^{MSADE}=\frac{\beta_{1}^{MSADE}}{\beta_{1}^{MSADE}+\beta_{2}^{MSADE}} \end{array}$$

and

$$\begin{array}{c} {\hat{R}}^{MSALDE}=\frac{\beta_{1}^{MSALDE}}{\beta_{1}^{MSALDE}+\beta_{2}^{MSALDE}}. \end{array}$$

### 2.6 Cramér-Von Mises estimation

The CMEs ([35, 36]) of the unknown parameters *β*_1_, *β*_2_ and *α* denoted by ${\hat{\beta_{1}}}^{CME},{\hat{\beta_{2}}}^{CME}$ and ${\hat{\alpha }}^{CME}$ are obtained by minimizing the following goodness-of-fit statistic

$$\begin{array}{ccc} CM & = & \frac{1}{12m}+\frac{1}{12r}+\sum_{i=1}^{m}{\left[\varphi_{1}\left(i,m\right)-e^{-\beta_{1}x_{i:m}^{\alpha }}\right]}^{2}+\sum_{j=1}^{r}{\left[\varphi_{2}\left(j,r\right)-e^{-\beta_{2}y_{j:r}^{\alpha }}\right]}^{2}, \end{array}$$

with respect to *β*_1_, *β*_2_ and *α*, where $\varphi_{1}\left(i,m\right)=\frac{2(m-i)+1}{2m}$ and $\varphi_{2}\left(j,r\right)=\frac{2(r-j)+1}{2r}$, where *CM* ≡ *CM*(*β*_1_, *β*_2_, *α*). These estimates can also be obtained by solving the following three equations simultaneously

$$\begin{array}{c} \frac{\partial CM}{\partial \beta_{1}}=\sum_{i=1}^{m}x_{i:m}^{\alpha }e^{-\beta_{1}y_{i:m}^{\alpha }}[\varphi_{1}\left(i,m\right)-e^{-\beta_{1}x_{i:m}^{\alpha }}]=0,\\ \frac{\partial CM}{\partial \beta_{2}}=\sum_{j=1}^{r}y_{j:r}^{\alpha }e^{-\beta_{2}y_{j:r}^{\alpha }}[\varphi_{2}\left(j,r\right)-e^{-\beta_{2}y_{j:r}^{\alpha }}]=0 \end{array}$$

and

$$\begin{array}{ccc} \frac{\partial CM}{\partial \alpha } & = & \beta_{1}\sum_{i=1}^{m}y_{i:m}^{\alpha }\;\text{log}\;\left(x_{i:m}\right)e^{-\beta_{1}x_{i:m}^{\alpha }}[\varphi_{1}\left(i,m\right)-e^{-\beta_{1}x_{i:m}^{\alpha }}]+\beta_{2}\sum_{j=1}^{r}y_{j:r}^{\alpha }\;\text{log}\;\left(y_{j:r}\right)\\ & \times & e^{-\beta_{1}y_{j:r}^{\alpha }}[\varphi_{2}\left(j,r\right)-e^{-\beta_{2}y_{j:r}^{\alpha }}]=0. \end{array}$$

The CME of *R* can be obtained by

$$\begin{array}{c} {\hat{R}}^{CME}=\frac{\beta_{1}^{CME}}{\beta_{1}^{CME}+\beta_{2}^{CME}}. \end{array}$$

### 2.7 Anderson-Darling and Right-tail Anderson-Darling estimation

The ADE is another type of minimum distance estimator and is obtained by minimizing an Anderson–Darling statistics. [37] introduced a modification of the Anderson–Darling statistics, namely Right-tail Anderson–Darling statistics. The ADEs of the unknown parameters *β*_1_, *β*_2_ and *α* denoted by ${\hat{\beta_{1}}}^{ADE},{\hat{\beta_{2}}}^{ADE}$ and ${\hat{\alpha }}^{ADE}$ are obtained by minimizing the following function

$$\begin{array}{ccc} A & = & -m-r-\frac{1}{m}\sum_{i=1}^{m}\left(2i-1\right)[\;\text{log}\;(1-e^{-\beta_{1}x_{i:m}^{\alpha }})-\beta_{1}yx_{m+1-i:m}^{\alpha }]-\frac{1}{r}\sum_{j=1}^{r}\left(2j-1\right)\\ & \times & [\;\text{log}\;(1-e^{-\beta_{2}y_{j:r}^{\alpha }})-\beta_{2}y_{r+1-j:r}^{\alpha }], \end{array}$$

with respect to *β*_1_, *β*_2_ and *α*, where *A* ≡ *A*(*β*_1_, *β*_2_, *α*). Equivalently, these estimates can also be obtained by solving the following three equations simultaneously

$$\begin{array}{c} \frac{\partial A}{\partial \beta_{1}}=\frac{1}{m}\sum_{i=1}^{m}\left(2i-1\right)(\frac{x_{i:m}^{\alpha }e^{-\beta_{1}x_{i:m}^{\alpha }}}{1-e^{-\beta_{1}x_{i:m}^{\alpha }}}-x_{m+1-i:m}^{\alpha })=0,\\ \frac{\partial A}{\partial \beta_{2}}=\frac{1}{r}\sum_{j=1}^{r}\left(2j-1\right)(\frac{z_{j:r}^{\alpha }e^{-\beta_{2}y_{j:r}^{\alpha }}}{1-e^{-\beta_{2}y_{j:r}^{\alpha }}}-y_{r+1-j:r}^{\alpha })=0 \end{array}$$

and

$$\begin{array}{ccc} \frac{\partial A}{\partial \alpha } & = & \frac{\beta_{1}}{m}\sum_{i=1}^{m}\left(2i-1\right)(\frac{x_{i:m}^{\alpha }\;\text{log}\;\left(x_{i:m}\right)e^{-\beta_{1}x_{i:m}^{\alpha }}}{1-e^{-\beta_{1}x_{i:m}^{\alpha }}}-x_{m+1-i:m}^{\alpha }\;\text{log}\;\left(x_{m+1-i:m}\right))\\ & + & \frac{\beta_{2}}{r}\sum_{j=1}^{r}\left(2j-1\right)(\frac{y_{j:r}^{\alpha }\;\text{log}\;\left(y_{j:r}\right)e^{-\beta_{2}y_{j:r}^{\alpha }}}{1-e^{-\beta_{2}y_{j:r}^{\alpha }}}-y_{r+1-j:r}^{\alpha }\;\text{log}\;\left(y_{r+1-j:r}\right))=0. \end{array}$$

Similarly, the RADEs of the unknown parameters *β*_1_, *β*_2_ and *α* denoted by ${\hat{\beta_{1}}}^{RADE},{\hat{\beta_{2}}}^{RADE}$ and ${\hat{\alpha }}^{RADE}$ can be obtained by minimizing the following function

$$\begin{array}{ccc} R & = & \frac{m+r}{2}-2\sum_{i=1}^{m}(1-e^{-\beta_{1}x_{i:m}^{\alpha }})-\frac{\beta_{1}}{m}\sum_{i=1}^{m}\left(2i-1\right)x_{m+1-i:m}^{\alpha }-2\sum_{j=1}^{r}(1-e^{-\beta_{2}y_{j:r}^{\alpha }})\\ & - & \frac{\beta_{2}}{r}\sum_{j=1}^{r}\left(2j-1\right)y_{r+1-j:r}^{\alpha }, \end{array}$$

with respect to *β*_1_, *β*_2_ and *α*, where *R* ≡ *R*(*β*_1_, *β*_2_, *α*), or by solving the following three equations simultaneously

$$\begin{array}{c} \frac{\partial R}{\partial \beta_{1}}=2\sum_{i=1}^{m}x_{i:m}^{\alpha }e^{-\beta_{1}x_{i:m}^{\alpha }}+\frac{1}{m}\sum_{i=1}^{m}\left(2i-1\right)x_{m+1-i:m}^{\alpha }=0,\\ \frac{\partial R}{\partial \beta_{2}}=2\sum_{j=1}^{r}y_{j:r}^{\alpha }e^{-\beta_{2}y_{j:r}^{\alpha }}+\frac{1}{r}\sum_{j=1}^{r}\left(2j-1\right)y_{r+1-j:r}^{\alpha }=0 \end{array}$$

and

$$\begin{array}{ccc} \frac{\partial R}{\partial \alpha } & = & 2\beta_{1}\sum_{i=1}^{m}y_{i:m}^{\alpha }\;\text{log}\;\left(y_{i:m}\right)e^{-\beta_{1}x_{i:m}^{\alpha }}+\frac{\beta_{1}}{m}\sum_{i=1}^{m}\left(2i-1\right)x_{m+1-i:m}^{\alpha }\;\text{log}\;\left(x_{m+1-i:m}\right)\\ & + & 2\beta_{2}\sum_{j=1}^{r}y_{j:r}^{\alpha }\;\text{log}\;\left(y_{j:r}\right)e^{-\beta_{2}y_{j:r}^{\alpha }}+\frac{\beta_{2}}{r}\sum_{j=1}^{r}\left(2j-1\right)y_{r+1-j:r}^{\alpha }\;\text{log}\;\left(y_{r+1-j:r}\right)=0. \end{array}$$

The ADE and RADE of *R* can be obtained, respectively, by

$$\begin{array}{c} {\hat{R}}^{ADE}=\frac{\beta_{1}^{ADE}}{\beta_{1}^{ADE}+\beta_{2}^{ADE}} \end{array}$$

and

$$\begin{array}{c} {\hat{R}}^{RADE}=\frac{\beta_{1}^{RADE}}{\beta_{1}^{RADE}+\beta_{2}^{RADE}}. \end{array}$$

## 3 Bootstrap confidence intervals

In this section, we propose the use of two confidence intervals for the parameters *β*_1_, *β*_2_ and *α* based on the parametric bootstrap methods. The two different parametric confidence intervals are (i) percentile bootstrap (Boot-P) and (ii) bias corrected percentile bootstrap (Boot-BCP) confidence intervals. The following steps illustrate briefly how to estimate the confidence intervals of *R*:

A) Boot-P confidence interval
  1 Generate the samples {*x*_1:*m*_, …, *x*_*m*:*m*_} and {*y*_1:*r*_, …, *y*_*r*:*r*_} and obtain the estimates ${\hat{\beta_{1}}}^{*}$, ${\hat{\beta_{2}}}^{*}$, ${\hat{\alpha }}^{*}$ and ${\hat{R}}^{*}$ from the original data, where ${\hat{\beta_{1}}}^{*}$, ${\hat{\beta_{2}}}^{*}$, ${\hat{\alpha }}^{*}$ and ${\hat{R}}^{*}$ are the estimates obtained from the different estimation methods.
  2 Use ${\hat{\beta_{1}}}^{*}$ and ${\hat{\alpha }}^{*}$ to generate a bootstrap sample $\left\{x_{1:m}^{b},...,x_{m:m}^{b}\right\}$ and ${\hat{\beta_{2}}}^{*}$ and ${\hat{\alpha }}^{*}$ to generate a bootstrap sample $\left\{y_{1:r}^{b},...,y_{r:r}^{b}\right\}$.
  3 Based on $\left\{x_{1:m}^{b},...,x_{m:m}^{b}\right\}$ and $\left\{y_{1:r}^{b},...,y_{r:r}^{b}\right\}$ obtain the bootstrap estimate of *R*, say ${\hat{R}}^{*b}$.
  4 Repeat step 1-3 *B* times to have ${\hat{R}}^{*b(1)},...,{\hat{R}}^{*b(B)}$.
  5 Arrange the bootstrap estimates in step 4 in ascending order as ${\hat{R}}^{*b[1]},...,{\hat{R}}^{*b[B]}$.
  6 The two-sided 100(1−*τ*) Boot-P confidence interval of *R* is given by
    $$\begin{array}{c} \{{\hat{R}}^{*b[B\tau /2]},{\hat{R}}^{*b[B(1-\tau /2)]}\}. \end{array}$$
B) Boot-BCP confidence interval
  1 Same as 1, 2, 3 and 4 in Boot-p.
  2 The two-sided 100(1−*τ*) Boot-BCP confidence intervals for the unknown parameters are given by
    $$\begin{array}{c} \{{\hat{R}}^{*b[B\delta_{1}]},{\hat{R}}^{*b[B\delta_{2}]}\}. \end{array}$$
    where
    $$\begin{array}{c} \delta_{1}=\Phi \left(2z_{0}+z_{\tau /2}\right)\;\;\;\text{and}\;\;\;\delta_{2}=\Phi \left(2z_{0}+z_{1-\tau /2}\right), \end{array}$$
    where Φ(.) is the CDF of the standard normal distribution and *z*_*τ*_ = Φ^−1^(*τ*) and *z*_0_ can be obtained as follows
    $$\begin{array}{c} z_{0}=\Phi^{-1}(\frac{\#\{{\hat{R}}^{*b(i)}<{\hat{R}}^{*}\}}{B}),i=1,...,B,. \end{array}$$

## 4 Simulation study

In this section an extensive Mont Carlo simulation study is performed to compare the finite sample properties of the different estimators discussed in the previous sections in terms of relative biases (RBs) and relative mean squared errors (RMSEs). We also compare two different bootstrap confidence intervals in terms confidence interval lengths (CILs). We consider the following parameter values; *β*_1_ = (0.5, 1, 1.5, 2), *β*_2_ = (0.5, 1, 1.5, 2, 2.5) and kept *α* constant at *α* = 1.5 in all the cases. We choose the following different sample sizes; (*m*, *r*) = (10, 10), (25, 25) and (50, 50). The results of all setting are obtained based on 1000 simulations. The bootstrap confidence intervals are obtained based on 1000 resampling with nominal level 0.95 in all the cases. We obtain the MLE of *α* using the same the iterative algorithm discussed in Kundu and Gupta [3], then we obtain the MLEs of *β*_1_ and *β*_2_ from (8). We use the MLEs of *β*_1_, *β*_2_ and *α* as an initial values to start the numerical solution for the other methods. The average RBs, RMSEs and CILs are obtained as

$$\begin{array}{c} RB_{k}=\frac{1}{1000}\sum_{t=1}^{1000}\frac{\left({\hat{R}}_{kt}^{*}-R\right)}{R},k=1,...,10, \end{array}$$

$$\begin{array}{c} RMSE_{k}=\frac{1}{1000}\sum_{t=1}^{1000}\frac{{\left({\hat{R}}_{kt}^{*}-R\right)}^{2}}{R},k=1,...,10, \end{array}$$

and

$$\begin{array}{c} CIL_{k}=\frac{1}{1000}\sum_{t=1}^{1000}\left(U_{kt}-L_{kt}\right),k=1,...,10, \end{array}$$

where *L*_*kt*_ and *U*_*kt*_ for *t* = 1, …, 1000 and *k* = 1, …, 10 are the lower and upper confidence interval bounds, respectively. For more details about the mean square error see for example [38] and [39]. The average RBs and RMSEs are displayed in Tables 1, 2 and 3, while the average CILs are displayed in Tables 4, 5 and 6. From these Tables, we conclude the following results:

1. As the sample size increases the RBs decrease and tend to zero which indicates the asymptotically unbiased property for the different estimates.
2. As the sample size increases the RMSEs decrease which indicates that the different estimates are consistent.
3. In most of the cases, the PCEs perform better than other estimates in terms of minimum RMSEs.
4. Similarly, the MSADEs have the largest RMSEs among other estimates in all the cases.
5. For the small sample size, the MPSEs perform better than the MLEs in most of the cases, and when the sample size increase they approximately have the same RMSEs.
6. As the sample size increases the CILs of the two bootstrap confidence intervals decrease in all the cases for all the estimation methods.
7. In most of the cases, the PCEs have the smallest CILs followed by the MPSEs.
8. In all the cases, the MSADEs have the largest CILs.
9. The Boot-BCP confidence intervals perform better than the Boot-P confidence intervals in most of the cases in terms of minimum CILs.

Based on the the above results, we can confirm the superiority of the PCEs followed by MPSEs to estimate the stress-strength parameter of the Weibull distributions with the same shape parameter. We can also conclude that the MPSEs are highly competitive with the MLEs in small as well as in large sample sizes. Since the MLEs have low efficiency for the small sample sizes, we suggest to use the MPSE method to estimate the stress-strength parameter of the Weibull distributions with high efficiency, especially for the small samples. Also, many researchers used the different proposed estimation methods to obtain only the point estimates without referring to the interval estimation. This is due to that no any theory in the literature to obtain the interval estimation of these methods except the MLE and MPSE methods. The simulation results show that the bootstrap confidence intervals can be used to achieve this objective.

**Table 1:** Average values of RBs (first row) and RMSEs (second reow) for (*m*, *r*) = (10, 10).

Initial values		Method									
*β*_1_	*β*_2_	MLEs	LSEs	WLSEs	PCEs	MPSEs	MSADEs	MSALDEs	CMEs	ADEs	RTADEs
0.5	0.5	0.0079	0.0050	0.0040	0.0056	0.0065	0.0068	0.0047	0.0052	0.0048	0.0088
		0.0298	0.0288	0.0289	0.0224	0.0294	0.0490	0.0397	0.0402	0.0271	0.0278
	1	0.0108	0.0612	0.0470	0.0584	0.0125	0.1807	0.0594	0.0014	0.0395	0.0191
		0.0358	0.0377	0.0378	0.0302	0.0356	0.0828	0.0517	0.0481	0.0336	0.0352
	1.5	0.0333	0.0928	0.0724	0.0901	0.0113	0.3302	0.0961	0.0254	0.0572	0.0275
		0.0358	0.0422	0.0409	0.0348	0.0376	0.1144	0.0552	0.0482	0.0373	0.0362
	2	0.0464	0.1358	0.1028	0.1232	0.0133	0.4482	0.1239	0.0194	0.0775	0.0302
		0.0381	0.0488	0.0457	0.0425	0.0396	0.1452	0.0613	0.0507	0.0417	0.0403
	2.5	0.0360	0.1731	0.1376	0.1664	0.0391	0.5699	0.1941	0.0355	0.1061	0.0519
		0.0333	0.0481	0.0445	0.0432	0.0373	0.1653	0.0636	0.0453	0.0394	0.0369
1	0.5	0.0209	0.0228	0.0154	0.0260	0.0091	0.0963	0.0238	0.0078	0.0188	0.0129
		0.0187	0.0182	0.0181	0.0148	0.0181	0.0406	0.0261	0.0231	0.0173	0.0177
	1	0.0097	0.0122	0.0126	0.0084	0.0118	0.0138	0.0122	0.0157	0.0096	0.0099
		0.0293	0.0274	0.0278	0.0222	0.0282	0.0488	0.0366	0.0377	0.0266	0.0273
	1.5	0.0145	0.0181	0.0138	0.0230	0.0046	0.0947	0.0198	0.0173	0.0175	0.0085
		0.0349	0.0327	0.0332	0.0276	0.0336	0.0664	0.0438	0.0438	0.0313	0.0332
	2	0.0181	0.0594	0.0471	0.0539	0.0112	0.1597	0.0420	0.0155	0.0387	0.0138
		0.0335	0.0345	0.0344	0.0278	0.0334	0.0771	0.0472	0.0436	0.0324	0.0320
	2.5	0.0446	0.0578	0.0401	0.0515	0.0093	0.2667	0.0628	0.0342	0.0266	0.0131
		0.0370	0.0392	0.0388	0.0325	0.0379	0.0963	0.0526	0.0486	0.0359	0.0362
1.5	0.5	0.0092	0.0316	0.0230	0.0306	0.0033	0.1093	0.0314	0.0065	0.0188	0.0083
		0.0127	0.0141	0.0136	0.0123	0.0127	0.0373	0.0187	0.0160	0.0127	0.0125
	1	0.0061	0.0142	0.0103	0.0180	0.0050	0.0683	0.0245	0.0091	0.0091	0.0053
		0.0245	0.0232	0.0236	0.0193	0.0238	0.0461	0.0315	0.0317	0.0224	0.0229
	1.5	0.0049	0.0073	0.0075	0.0059	0.0039	0.0177	0.0064	0.0067	0.0065	0.0060
		0.0280	0.0271	0.0273	0.0209	0.0274	0.0543	0.0372	0.0369	0.0256	0.0259
	2	0.0175	0.0156	0.0128	0.0190	0.0155	0.0484	0.0196	0.0093	0.0099	0.0143
		0.0322	0.0296	0.0307	0.0251	0.0305	0.0587	0.0418	0.0402	0.0288	0.0301
	2.5	0.0199	0.0300	0.0209	0.0263	0.0038	0.1042	0.0331	0.0124	0.0156	0.0021
		0.0355	0.0356	0.0356	0.0286	0.0349	0.0679	0.0470	0.0467	0.0337	0.0338
2	0.5	0.0102	0.0366	0.0285	0.0312	0.0051	0.1267	0.0382	0.0021	0.0211	0.0081
		0.0090	0.0116	0.0110	0.0101	0.0095	0.0381	0.0161	0.0118	0.0099	0.0096
	1	0.0120	0.0244	0.0179	0.0208	0.0064	0.1020	0.0281	0.0073	0.0144	0.0122
		0.0178	0.0174	0.0175	0.0148	0.0176	0.0423	0.0240	0.0220	0.0166	0.0169
	1.5	0.0057	0.0243	0.0211	0.0212	0.0127	0.0576	0.0236	0.0088	0.0180	0.0124
		0.0264	0.0256	0.0264	0.0203	0.0259	0.0487	0.0332	0.0350	0.0238	0.0248
	2	0.0098	0.0088	0.0082	0.0092	0.0079	0.0105	0.0022	0.0109	0.0094	0.0100
		0.0302	0.0274	0.0277	0.0222	0.0291	0.0505	0.0363	0.0382	0.0270	0.0278
	2.5	0.0084	0.0117	0.0078	0.0198	0.0042	0.0403	0.0210	0.0060	0.0080	0.0069
		0.0339	0.0322	0.0326	0.0257	0.0332	0.0607	0.0442	0.0446	0.0308	0.0321

**Table 2:** Average values of RBs (first row) and RMSEs (second row) for (*m*, *r*) = (25, 25).

Initial values		Method									
*β*_1_	*β*_2_	MLEs	LSEs	WLSEs	PCEs	MPSEs	MSADEs	MSALDEs	CMEs	ADEs	RTADEs
0.5	0.5	0.0015	0.0012	0.0021	0.0014	0.0020	0.0135	0.0024	0.0012	0.0022	0.0009
		0.0110	0.0122	0.0119	0.0094	0.0109	0.0366	0.0157	0.0138	0.0113	0.0109
	1	0.0019	0.0270	0.0165	0.0350	0.0057	0.1545	0.0296	0.0005	0.0181	0.0096
		0.0138	0.0167	0.0160	0.0130	0.0140	0.0568	0.0208	0.0182	0.0151	0.0145
	1.5	0.0062	0.0567	0.0365	0.0647	0.0121	0.3196	0.0703	0.0097	0.0377	0.0249
		0.0148	0.0185	0.0171	0.0157	0.0150	0.0857	0.0243	0.0190	0.0165	0.0156
	2	0.0332	0.0455	0.0187	0.0572	0.0114	0.4108	0.0529	0.0188	0.0183	0.0011
		0.0132	0.0165	0.0152	0.0157	0.0133	0.1036	0.0217	0.0167	0.0143	0.0142
	2.5	0.0319	0.0573	0.0273	0.0861	0.0075	0.5269	0.0812	0.0213	0.0298	0.0142
		0.0122	0.0179	0.0159	0.0169	0.0130	0.1173	0.0228	0.0176	0.0149	0.0139
1	0.5	0.0047	0.0179	0.0125	0.0236	0.0080	0.0945	0.0224	0.0048	0.0140	0.0104
		0.0068	0.0081	0.0078	0.0064	0.0068	0.0324	0.0099	0.0087	0.0073	0.0071
	1	0.0046	0.0069	0.0070	0.0035	0.0056	0.0269	0.0103	0.0072	0.0072	0.0057
		0.0113	0.0131	0.0126	0.0097	0.0112	0.0376	0.0150	0.0150	0.0120	0.0113
	1.5	0.0036	0.0181	0.0123	0.0166	0.0040	0.0857	0.0182	0.0046	0.0117	0.0077
		0.0135	0.0150	0.0147	0.0118	0.0134	0.0515	0.0193	0.0168	0.0139	0.0135
	2	0.0148	0.0139	0.0037	0.0229	0.0083	0.1518	0.0192	0.0129	0.0051	0.0026
		0.0137	0.0170	0.0162	0.0126	0.0141	0.0639	0.0212	0.0189	0.0151	0.0144
	2.5	0.0087	0.0320	0.0173	0.0439	0.0036	0.2451	0.0370	0.0059	0.0190	0.0101
		0.0144	0.0171	0.0159	0.0144	0.0145	0.0761	0.0218	0.0184	0.0155	0.0151
1.5	0.5	0.0075	0.0099	0.0042	0.0150	0.0027	0.1073	0.0141	0.0061	0.0044	0.0003
		0.0047	0.0058	0.0054	0.0049	0.0047	0.0285	0.0071	0.0061	0.0051	0.0049
	1	0.0006	0.0116	0.0079	0.0137	0.0041	0.0671	0.0153	0.0024	0.0091	0.0049
		0.0081	0.0093	0.0089	0.0071	0.0081	0.0311	0.0118	0.0104	0.0085	0.0082
	1.5	0.0048	0.0062	0.0064	0.0042	0.0057	0.0088	0.0030	0.0066	0.0057	0.0054
		0.0097	0.0115	0.0111	0.0081	0.0099	0.0342	0.0142	0.0132	0.0106	0.0097
	2	0.0167	0.0056	0.0088	0.0013	0.0140	0.0388	0.0112	0.0157	0.0090	0.0123
		0.0130	0.0143	0.0141	0.0112	0.0128	0.0456	0.0172	0.0162	0.0132	0.0130
	2.5	0.0078	0.0097	0.0032	0.0173	0.0034	0.0867	0.0125	0.0088	0.0034	0.0015
		0.0134	0.0160	0.0152	0.0122	0.0134	0.0503	0.0186	0.0180	0.0145	0.0138
2	0.5	0.0018	0.0146	0.0085	0.0206	0.0022	0.1199	0.0194	0.0015	0.0095	0.0054
		0.0036	0.0046	0.0042	0.0042	0.0037	0.0300	0.0060	0.0046	0.0039	0.0038
	1	0.0010	0.0174	0.0124	0.0190	0.0053	0.0909	0.0209	0.0043	0.0132	0.0076
		0.0068	0.0083	0.0079	0.0065	0.0068	0.0321	0.0103	0.0090	0.0074	0.0070
	1.5	0.0020	0.0047	0.0023	0.0082	0.0006	0.0414	0.0068	0.0024	0.0024	0.0006
		0.0092	0.0099	0.0097	0.0082	0.0090	0.0334	0.0124	0.0112	0.0093	0.0090
	2	0.0005	0.0029	0.0028	0.0003	0.0009	0.0081	0.0015	0.0031	0.0022	0.0011
		0.0109	0.0116	0.0114	0.0095	0.0107	0.0347	0.0144	0.0131	0.0109	0.0106
	2.5	0.0014	0.0104	0.0076	0.0116	0.0036	0.0285	0.0048	0.0037	0.0070	0.0057
		0.0112	0.0131	0.0128	0.0095	0.0112	0.0384	0.0164	0.0149	0.0121	0.0113

**Table 3:** Average values of RBs (first row) and RMSEs (second row) for (*m*, *r*) = (50, 50).

Initial values		Method									
*β*_1_	*β*_2_	MLEs	LSEs	WLSEs	PCEs	MPSEs	MSADEs	MSALDEs	CMEs	ADEs	RTADEs
0.5	0.5	0.0013	0.0010	0.0018	0.0008	0.0016	0.0069	0.0020	0.0010	0.0018	0.0006
		0.0051	0.0062	0.0058	0.0047	0.0051	0.0267	0.0073	0.0067	0.0056	0.0053
	1	0.0013	0.0029	0.0039	0.0116	0.0015	0.1365	0.0084	0.0003	0.0020	0.0072
		0.0061	0.0078	0.0072	0.0058	0.0062	0.0467	0.0091	0.0083	0.0070	0.0065
	1.5	0.0060	0.0229	0.0088	0.0375	0.0038	0.2745	0.0277	0.0013	0.0133	0.0019
		0.0067	0.0086	0.0078	0.0072	0.0068	0.0643	0.0104	0.0087	0.0075	0.0074
	2	0.0097	0.0321	0.0140	0.0513	0.0012	0.3952	0.0429	0.0005	0.0181	0.0010
		0.0072	0.0092	0.0082	0.0086	0.0073	0.0859	0.0115	0.0092	0.0081	0.0079
	2.5	0.0193	0.0210	0.0001	0.0502	0.0051	0.4932	0.0458	0.0187	0.0087	0.0012
		0.0061	0.0085	0.0074	0.0081	0.0062	0.1006	0.0100	0.0085	0.0070	0.0070
1	0.5	0.0039	0.0010	0.0028	0.0052	0.0054	0.0825	0.0049	0.0035	0.0011	0.0038
		0.0030	0.0039	0.0035	0.0029	0.0030	0.0234	0.0048	0.0041	0.0034	0.0032
	1	0.0027	0.0062	0.0054	0.0014	0.0029	0.0044	0.0010	0.0065	0.0051	0.0038
		0.0048	0.0060	0.0056	0.0044	0.0048	0.0257	0.0071	0.0064	0.0053	0.0049
	1.5	0.0018	0.0099	0.0065	0.0127	0.0017	0.0823	0.0086	0.0029	0.0068	0.0042
		0.0064	0.0073	0.0069	0.0056	0.0061	0.0356	0.0085	0.0077	0.0066	0.0063
	2	0.0005	0.0135	0.0027	0.0213	0.0018	0.1483	0.0169	0.0001	0.0043	0.0018
		0.0064	0.0084	0.0077	0.0064	0.0064	0.0457	0.0091	0.0088	0.0074	0.0069
	2.5	0.0041	0.0153	0.0058	0.0310	0.0004	0.2340	0.0209	0.0040	0.0097	0.0048
		0.0065	0.0085	0.0078	0.0068	0.0065	0.0598	0.0102	0.0088	0.0075	0.0071
1.5	0.5	0.0006	0.0079	0.0038	0.0143	0.0010	0.1013	0.0106	0.0002	0.0041	0.0002
		0.0024	0.0029	0.0027	0.0026	0.0023	0.0230	0.0034	0.0030	0.0026	0.0025
	1	0.0004	0.0054	0.0027	0.0076	0.0001	0.0580	0.0039	0.0007	0.0034	0.0018
		0.0041	0.0049	0.0047	0.0038	0.0041	0.0252	0.0061	0.0052	0.0046	0.0042
	1.5	0.0044	0.0055	0.0052	0.0041	0.0041	0.0086	0.0024	0.0056	0.0052	0.0046
		0.0049	0.0060	0.0056	0.0046	0.0049	0.0291	0.0074	0.0064	0.0054	0.0050
	2	0.0033	0.0051	0.0059	0.0011	0.0046	0.0343	0.0111	0.0035	0.0065	0.0061
		0.0055	0.0068	0.0063	0.0051	0.0054	0.0303	0.0084	0.0072	0.0061	0.0056
	2.5	0.0006	0.0056	0.0022	0.0164	0.0031	0.0765	0.0115	0.0064	0.0028	0.0013
		0.0059	0.0076	0.0070	0.0057	0.0059	0.0381	0.0087	0.0080	0.0067	0.0062
2	0.5	0.0011	0.0081	0.0035	0.0134	0.0001	0.1080	0.0124	0.0001	0.0047	0.0029
		0.0016	0.0023	0.0020	0.0020	0.0017	0.0226	0.0028	0.0023	0.0019	0.0019
	1	0.0010	0.0035	0.0004	0.0095	0.0018	0.0833	0.0078	0.0034	0.0017	0.0001
		0.0033	0.0043	0.0040	0.0032	0.0034	0.0254	0.0049	0.0045	0.0038	0.0036
	1.5	0.0001	0.0044	0.0021	0.0070	0.0005	0.0347	0.0047	0.0009	0.0021	0.0002
		0.0046	0.0054	0.0051	0.0043	0.0046	0.0252	0.0064	0.0057	0.0049	0.0047
	2	0.0015	0.0025	0.0024	0.0002	0.0019	0.0061	0.0012	0.0031	0.0021	0.0008
		0.0004	0.0062	0.0058	0.0046	0.0050	0.0282	0.0075	0.0066	0.0055	0.0052
	2.5	0.0012	0.0015	0.0005	0.0039	0.0015	0.0210	0.0007	0.0021	0.0003	0.0007
		0.0061	0.0071	0.0069	0.0056	0.0062	0.0321	0.0089	0.0076	0.0066	0.0063

**Table 4:** Average CILs of Boot-P (first row) and Boot-BCP (second row) for (*m*, *r*) = (10, 10).

Initial values		Method									
*β*_1_	*β*_2_	MLEs	LSEs	WLSEs	PCEs	MPSEs	MSADEs	MSALDEs	CMEs	ADEs	RTADEs
0.5	0.5	0.4798	0.4569	0.4715	0.4199	0.4572	0.5589	0.4683	0.4886	0.4357	0.4414
		0.4213	0.4462	0.4615	0.4194	0.4194	0.3541	0.4501	0.5106	0.4383	0.4915
	1	0.4708	0.4046	0.3736	0.3716	0.3028	0.5141	0.3574	0.3873	0.3412	0.3429
		0.3564	0.3732	0.3674	0.3578	0.3771	0.3007	0.3473	0.4135	0.3408	0.3444
	1.5	0.4884	0.3794	0.3735	0.3935	0.4126	0.5924	0.4140	0.3005	0.4148	0.4227
		0.4699	0.3828	0.3648	0.3932	0.3947	0.5844	0.3918	0.3206	0.4134	0.4080
	2	0.4686	0.3733	0.3753	0.3246	0.3375	0.5974	0.4373	0.3596	0.3323	0.3287
		0.4360	0.3435	0.3666	0.3037	0.3218	0.5706	0.4177	0.3831	0.3272	0.3236
	2.5	0.4813	0.2820	0.2689	0.2877	0.2658	0.4783	0.3057	0.2202	0.2860	0.2843
		0.4050	0.2452	0.2457	0.2784	0.2950	0.2374	0.2597	0.2613	0.2835	0.3120
1	0.5	0.5006	0.4634	0.4636	0.4133	0.4443	0.5541	0.5483	0.5323	0.4240	0.4505
		0.4839	0.4547	0.4551	0.4226	0.4141	0.4110	0.5469	0.5362	0.4261	0.4388
	1	0.4738	0.4578	0.4576	0.4064	0.4616	0.6079	0.5176	0.5360	0.4431	0.4558
		0.4044	0.4563	0.4624	0.4073	0.4124	0.5915	0.5176	0.5360	0.4429	0.4558
	1.5	0.4655	0.4645	0.4732	0.4149	0.4530	0.6257	0.5221	0.5470	0.4282	0.4337
		0.4771	0.4674	0.4673	0.4131	0.4131	0.6000	0.5233	0.5556	0.4282	0.4411
	2	0.5015	0.4371	0.4366	0.3674	0.3991	0.6451	0.4951	0.4599	0.4024	0.3850
		0.4476	0.4349	0.4436	0.3719	0.3828	0.6366	0.4799	0.4837	0.4001	0.3710
	2.5	0.4616	0.4471	0.4536	0.3858	0.4426	0.6202	0.4845	0.5260	0.4330	0.4489
		0.4514	0.4471	0.4545	0.3895	0.3889	0.6219	0.4727	0.5219	0.4363	0.4301
1.5	0.5	0.4884	0.2619	0.2087	0.3787	0.3636	0.5192	0.4688	0.1064	0.3377	0.3520
		0.4652	0.2126	0.1665	0.3698	0.3878	0.3411	0.4564	0.1398	0.3310	0.3541
	1	0.4686	0.4650	0.4579	0.4213	0.4596	0.6011	0.5283	0.5411	0.4388	0.4489
		0.4501	0.4615	0.4598	0.4177	0.4213	0.5944	0.5266	0.5446	0.4394	0.4496
	1.5	0.4707	0.4528	0.4590	0.4016	0.4622	0.6059	0.5295	0.5264	0.4403	0.4534
		0.4153	0.4497	0.4581	0.4088	0.4027	0.6081	0.5332	0.5284	0.4337	0.4539
	2	0.4819	0.4644	0.4699	0.4253	0.4754	0.6342	0.5275	0.5360	0.4579	0.4524
		0.4804	0.4566	0.4580	0.4270	0.4253	0.5488	0.5261	0.5358	0.4399	0.5039
	2.5	0.4761	0.4066	0.4240	0.3650	0.4221	0.6238	0.4030	0.4211	0.4053	0.4069
		0.4755	0.3977	0.4156	0.3747	0.3655	0.5974	0.3938	0.4425	0.4053	0.3773
2	0.5	0.4729	0.2804	0.2431	0.2648	0.1939	0.4532	0.3813	0.2152	0.2335	0.2246
		0.4303	0.2339	0.2175	0.2321	0.2708	0.1956	0.3557	0.2496	0.2202	0.1995
	1	0.4695	0.4659	0.4679	0.4344	0.4644	0.6694	0.5148	0.5437	0.4460	0.4580
		0.4585	0.4682	0.4732	0.4412	0.4369	0.6714	0.5088	0.5437	0.4493	0.4486
	1.5	0.4650	0.4318	0.4315	0.3887	0.4055	0.6034	0.4990	0.5043	0.4074	0.4242
		0.4498	0.4261	0.4223	0.3815	0.3899	0.5992	0.4996	0.5037	0.4089	0.3832
	2	0.4851	0.4482	0.4522	0.4163	0.4431	0.6372	0.4655	0.5067	0.4317	0.4395
		0.4400	0.4547	0.4481	0.4041	0.4280	0.5621	0.4570	0.5072	0.4298	0.4425
	2.5	0.4677	0.4450	0.4588	0.4040	0.4539	0.6181	0.5056	0.5334	0.4301	0.4448
		0.4658	0.4587	0.4707	0.4085	0.4115	0.5731	0.5056	0.5403	0.4353	0.4446

**Table 5:** Average CILs of Boot-P (first row) and Boot-BCP (second row) for (*m*, *r*) = (25, 25).

Initial values		Method									
*β*_1_	*β*_2_	MLEs	LSEs	WLSEs	PCEs	MPSEs	MSADEs	MSALDEs	CMEs	ADEs	RTADEs
0.5	0.5	0.2928	0.3057	0.2974	0.2672	0.2804	0.5256	0.3180	0.3242	0.2954	0.2876
		0.2746	0.3038	0.2963	0.2734	0.2689	0.4654	0.3118	0.3234	0.2954	0.2857
	1	0.2837	0.2997	0.2943	0.2645	0.2804	0.4266	0.3248	0.3222	0.2856	0.2854
		0.3032	0.2932	0.2938	0.2632	0.2693	0.3653	0.3248	0.3222	0.2872	0.3021
	1.5	0.2980	0.2433	0.2361	0.2423	0.2193	0.4300	0.2729	0.2294	0.2227	0.2118
		0.2462	0.2319	0.2324	0.2291	0.2430	0.2307	0.2608	0.2360	0.2198	0.2325
	2	0.2808	0.1914	0.1796	0.1865	0.1441	0.4210	0.1854	0.1771	0.1659	0.1620
		0.2494	0.1838	0.1711	0.1892	0.1816	0.2419	0.1743	0.1830	0.1661	0.1707
	2.5	0.2860	0.2164	0.2082	0.2286	0.1882	0.5248	0.2676	0.2138	0.1996	0.1987
		0.2108	0.2078	0.2075	0.2179	0.2271	0.4658	0.2632	0.2161	0.1975	0.1960
1	0.5	0.3103	0.2826	0.2733	0.2497	0.2409	0.5447	0.2925	0.2878	0.2659	0.2678
		0.3058	0.2740	0.2605	0.2383	0.2545	0.4338	0.2913	0.2921	0.2584	0.2143
	1	0.2932	0.2934	0.2894	0.2735	0.2914	0.5353	0.3304	0.3152	0.2808	0.2866
		0.2917	0.2934	0.2900	0.2769	0.2755	0.5313	0.3304	0.3142	0.2838	0.2846
	1.5	0.2849	0.2921	0.2925	0.2549	0.2790	0.5207	0.3293	0.3063	0.2789	0.2774
		0.2745	0.2950	0.2889	0.2530	0.2552	0.5137	0.3295	0.3095	0.2830	0.2771
	2	0.2902	0.2900	0.2865	0.2645	0.2775	0.5263	0.3143	0.3046	0.2790	0.2804
		0.2719	0.2999	0.2855	0.2579	0.2645	0.4842	0.3252	0.3060	0.2755	0.2729
	2.5	0.2778	0.2798	0.2748	0.2370	0.2479	0.5427	0.3231	0.2900	0.2633	0.2556
		0.2625	0.2744	0.2746	0.2339	0.2387	0.4984	0.3228	0.3017	0.2664	0.2540
1.5	0.5	0.2980	0.3151	0.3093	0.2701	0.2783	0.5589	0.3347	0.3313	0.2954	0.2843
		0.2853	0.3170	0.3057	0.2709	0.2721	0.5201	0.3272	0.3264	0.2903	0.2804
	1	0.2808	0.3013	0.2990	0.2613	0.2846	0.5354	0.3366	0.3208	0.2878	0.2804
		0.2780	0.3088	0.3025	0.2615	0.2614	0.5446	0.3356	0.3247	0.2846	0.2794
	1.5	0.2815	0.2861	0.2890	0.2660	0.2781	0.5333	0.3368	0.3043	0.2804	0.2817
		0.2865	0.2879	0.2831	0.2641	0.2652	0.4831	0.3357	0.3035	0.2817	0.2777
	2	0.2897	0.2825	0.2706	0.2412	0.2539	0.5351	0.2866	0.2904	0.2642	0.2500
		0.0886	0.2866	0.2773	0.2414	0.2356	0.5365	0.2861	0.3152	0.2673	0.2741
	2.5	0.2881	0.2769	0.2706	0.2604	0.2535	0.4474	0.3087	0.2892	0.2594	0.2568
		0.0693	0.2685	0.2631	0.2484	0.2615	0.3342	0.3170	0.2894	0.2528	0.2641
2	0.5	0.2930	0.1935	0.1892	0.1859	0.1824	0.4874	0.2153	0.1748	0.1978	0.1921
		0.2747	0.1774	0.1792	0.1756	0.1874	0.3837	0.1866	0.1791	0.1842	0.0973
	1	0.2860	0.2838	0.2769	0.2548	0.2656	0.4539	0.3302	0.2980	0.2806	0.2625
		0.2771	0.2843	0.2780	0.2632	0.2562	0.2750	0.3331	0.2949	0.2762	0.2611
	1.5	0.2905	0.3016	0.2970	0.2555	0.2752	0.5782	0.3276	0.3181	0.2860	0.2748
		0.2715	0.3069	0.3021	0.2569	0.2542	0.5559	0.3226	0.3220	0.2886	0.2766
	2	0.2739	0.2928	0.2912	0.2543	0.2705	0.4925	0.3269	0.3116	0.2869	0.2698
		0.2620	0.2928	0.2869	0.2582	0.2550	0.3536	0.3209	0.3155	0.2852	0.2703
	2.5	0.3026	0.3101	0.3062	0.2769	0.2937	0.4902	0.3484	0.3292	0.3028	0.2959
		0.3016	0.3118	0.3076	0.2773	0.2785	0.4357	0.3449	0.3260	0.3007	0.2945

**Table 6:** Average CILs of Boot-P (first row) and Boot-BCP (second row) for (*m*, *r*) = (50, 50).

Initial values		Method									
*β*_1_	*β*_2_	MLEs	LSEs	WLSEs	PCEs	MPSEs	MSADEs	MSALDEs	CMEs	ADEs	RTADEs
0.5	0.5	0.1958	0.2192	0.2079	0.1907	0.1976	0.4731	0.2339	0.2253	0.2021	0.2007
		0.1810	0.2190	0.2069	0.1888	0.1897	0.4736	0.2339	0.2254	0.2020	0.1932
	1	0.1972	0.2097	0.2049	0.1857	0.1917	0.4896	0.2284	0.2153	0.2015	0.1947
		0.1824	0.2089	0.2040	0.1798	0.1837	0.4772	0.2284	0.2143	0.2003	0.1905
	1.5	0.2003	0.1962	0.1849	0.1707	0.1685	0.4739	0.2168	0.1975	0.1811	0.1770
		0.1946	0.1931	0.1859	0.1788	0.1703	0.4027	0.2089	0.1976	0.1821	0.1780
	2	0.2032	0.1854	0.1775	0.1705	0.1533	0.4907	0.1996	0.1865	0.1722	0.1718
		0.1759	0.1839	0.1799	0.1734	0.1689	0.4453	0.1898	0.1875	0.1756	0.1811
	2.5	0.1960	0.1615	0.1503	0.1493	0.1348	0.4177	0.1708	0.1599	0.1479	0.1443
		0.1634	0.1535	0.1483	0.1418	0.1493	0.3016	0.1583	0.1599	0.1471	0.1182
1	0.5	0.2001	0.2216	0.2124	0.1884	0.1942	0.4637	0.2221	0.2272	0.2000	0.2039
		0.1942	0.2155	0.2120	0.1804	0.1871	0.4642	0.2203	0.2272	0.2046	0.1969
	1	0.1992	0.2232	0.2107	0.1909	0.2011	0.5187	0.2389	0.2315	0.2081	0.2039
		0.1990	0.2208	0.2141	0.1883	0.1904	0.5133	0.2390	0.2311	0.2062	0.2054
	1.5	0.1954	0.1853	0.1785	0.1632	0.1645	0.4923	0.1817	0.1872	0.1750	0.1689
		0.1838	0.1785	0.1785	0.1689	0.1632	0.4919	0.1865	0.1874	0.1754	0.1687
	2	0.2029	0.2214	0.2079	0.1915	0.1891	0.4871	0.2299	0.2283	0.2055	0.1952
		0.2004	0.2205	0.2079	0.1882	0.1932	0.4749	0.2291	0.2284	0.2046	0.1972
	2.5	0.2030	0.1831	0.1676	0.1699	0.1550	0.4567	0.1888	0.1840	0.1693	0.1676
		0.1912	0.1793	0.1676	0.1621	0.1713	0.3688	0.1887	0.1848	0.1693	0.1636
1.5	0.5	0.2003	0.1879	0.1816	0.1699	0.1645	0.4763	0.1956	0.1905	0.1754	0.1666
		0.0103	0.1896	0.1802	0.1764	0.1699	0.4538	0.1942	0.1924	0.1718	0.1728
	1	0.2056	0.2284	0.2157	0.1980	0.2031	0.4936	0.2383	0.2359	0.2091	0.2057
		0.1998	0.2275	0.2171	0.1952	0.1984	0.4846	0.2389	0.2368	0.2086	0.2046
	1.5	0.1931	0.2229	0.2147	0.1903	0.1909	0.4961	0.2323	0.2299	0.2100	0.1997
		0.1884	0.2258	0.2121	0.1902	0.1872	0.4764	0.2299	0.2290	0.2061	0.1983
	2	0.2001	0.2151	0.2124	0.1844	0.1946	0.4817	0.2305	0.2211	0.2078	0.1993
		0.1936	0.2209	0.2119	0.1843	0.1843	0.4777	0.2322	0.2219	0.2057	0.1989
	2.5	0.2033	0.2198	0.2081	0.1851	0.1908	0.5100	0.2450	0.2263	0.2036	0.1947
		0.1092	0.2154	0.2091	0.1823	0.1829	0.5059	0.2448	0.2244	0.2050	0.1949
2	0.5	0.1951	0.1707	0.1622	0.1579	0.1479	0.4515	0.1934	0.1694	0.1556	0.1548
		0.1709	0.1673	0.1628	0.1517	0.1592	0.4005	0.1835	0.1687	0.1570	0.1455
	1	0.2031	0.2039	0.1982	0.1774	0.1822	0.4819	0.2241	0.2083	0.1989	0.1925
		0.0220	0.2031	0.1982	0.1783	0.1813	0.4872	0.2159	0.2135	0.1987	0.1835
	1.5	0.2084	0.2169	0.2099	0.1955	0.2024	0.5036	0.2392	0.2241	0.2091	0.2051
		0.2054	0.2169	0.2099	0.1953	0.1961	0.4899	0.2444	0.2227	0.2076	0.1977
	2	0.1931	0.2159	0.2075	0.1880	0.1951	0.4734	0.2410	0.2227	0.2037	0.1944
		0.1902	0.2158	0.2093	0.1882	0.1941	0.3132	0.2410	0.2263	0.2022	0.2183
	2.5	0.1909	0.2083	0.2055	0.1800	0.1925	0.4627	0.2237	0.2139	0.2001	0.1930
		0.1934	0.2094	0.2061	0.1799	0.1793	0.3176	0.2286	0.2161	0.2037	0.1963

## 5 Data analysis

In this section, we analyze a data set of the strength data originally reported by [40]. The data represent the strength data measured in GPA, for single carbon fibers and impregnated 1000-carbon fiber tows. Single fibers were tested under tension at gauge lengths of 1, 10, 20, and 50mm. Impregnated tows of 1000 fibers were tested at gauge lengths of 20, 50, 150 and 300 mm. [3] analyzed these data by considering the single fibers of 20 mm as data set I (DSI) and 10 mm as data set II (DSII) in gauge length. The two data sets are given below.

**Fig 1:**
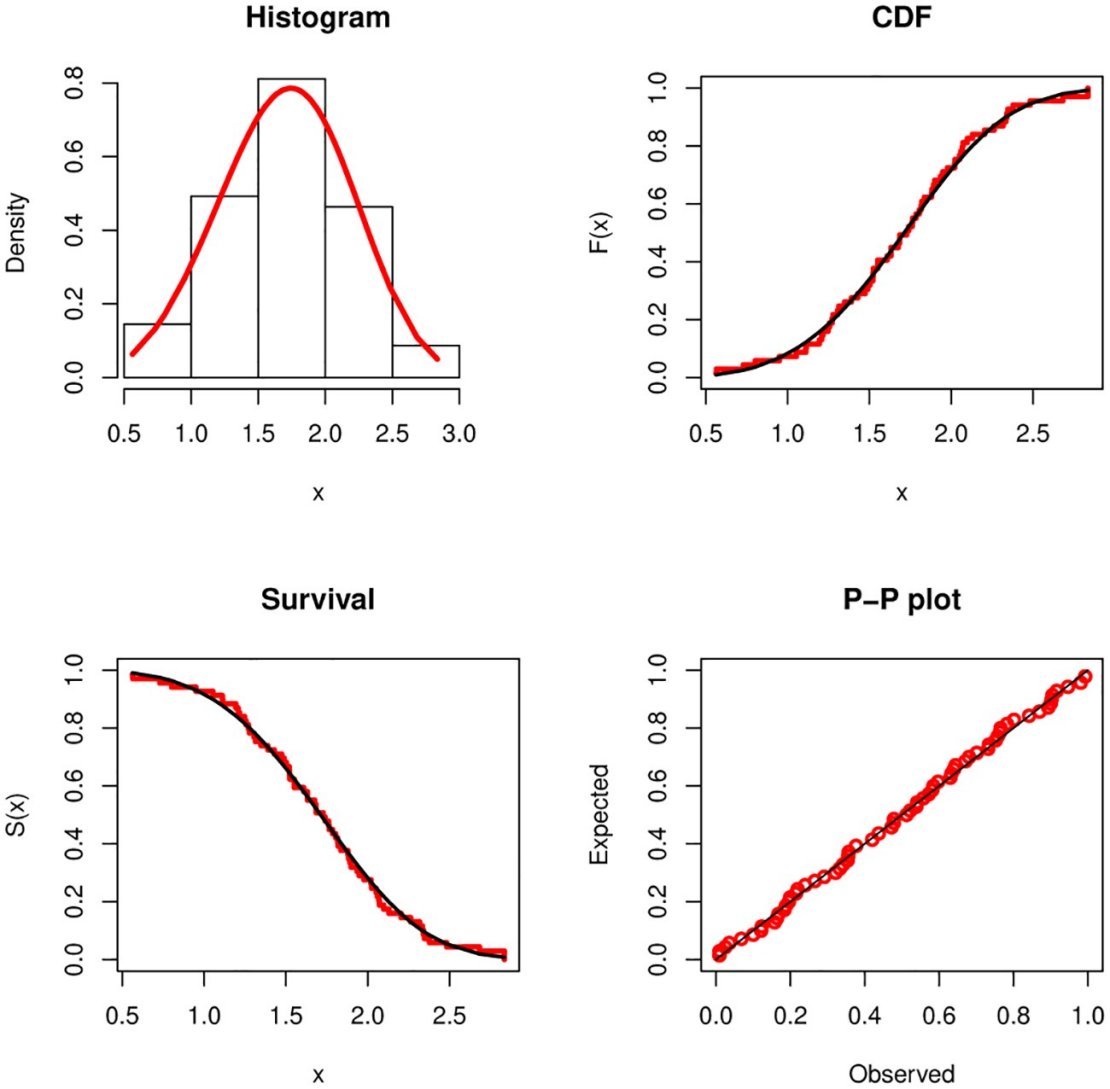
Fitted density, estimated CDF, survival function and P-P plots for DSI.

**Fig 2:**
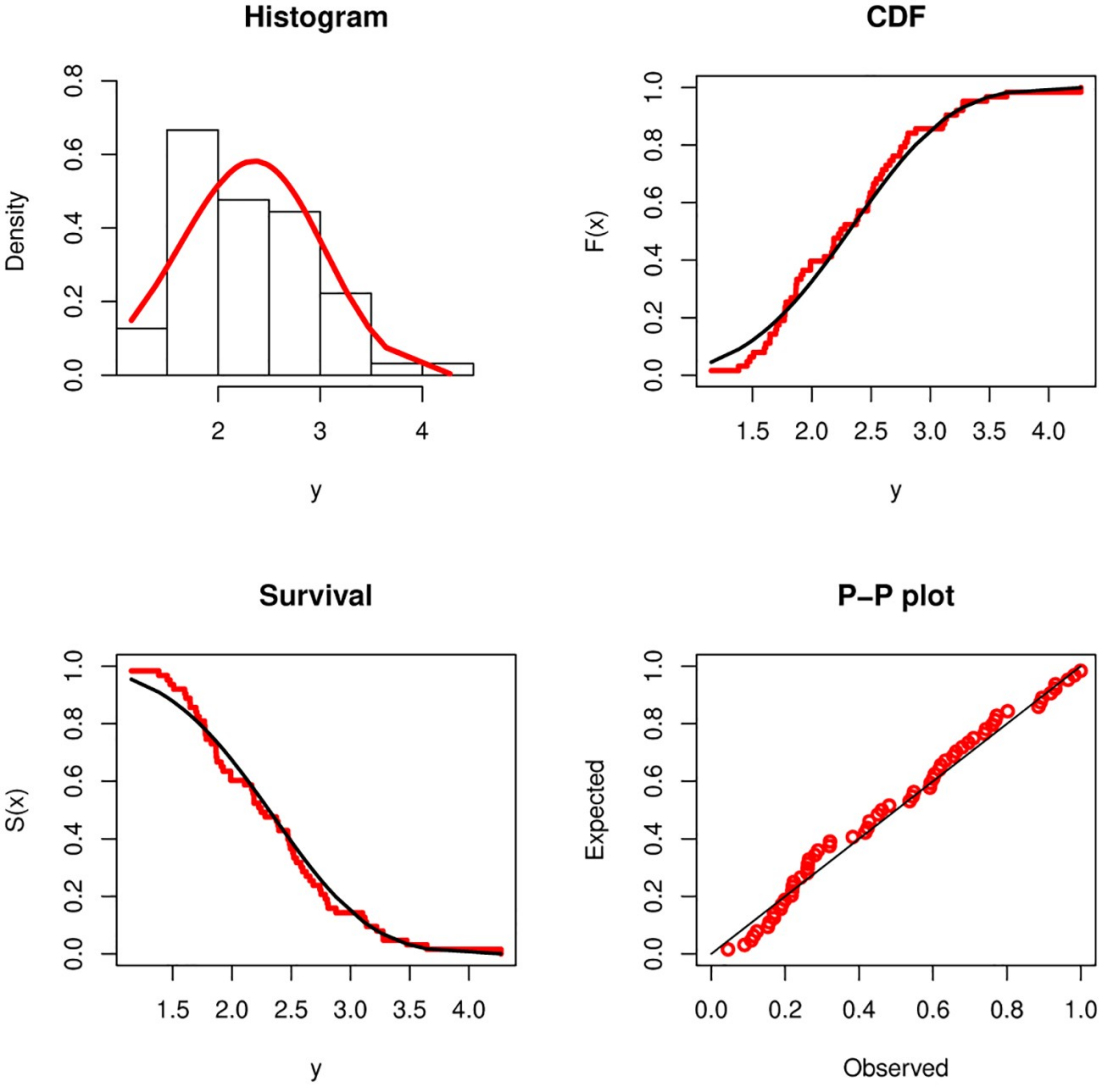
Fitted density, estimated CDF, survival function and P-P plots for DSII.

**Fig 3:**
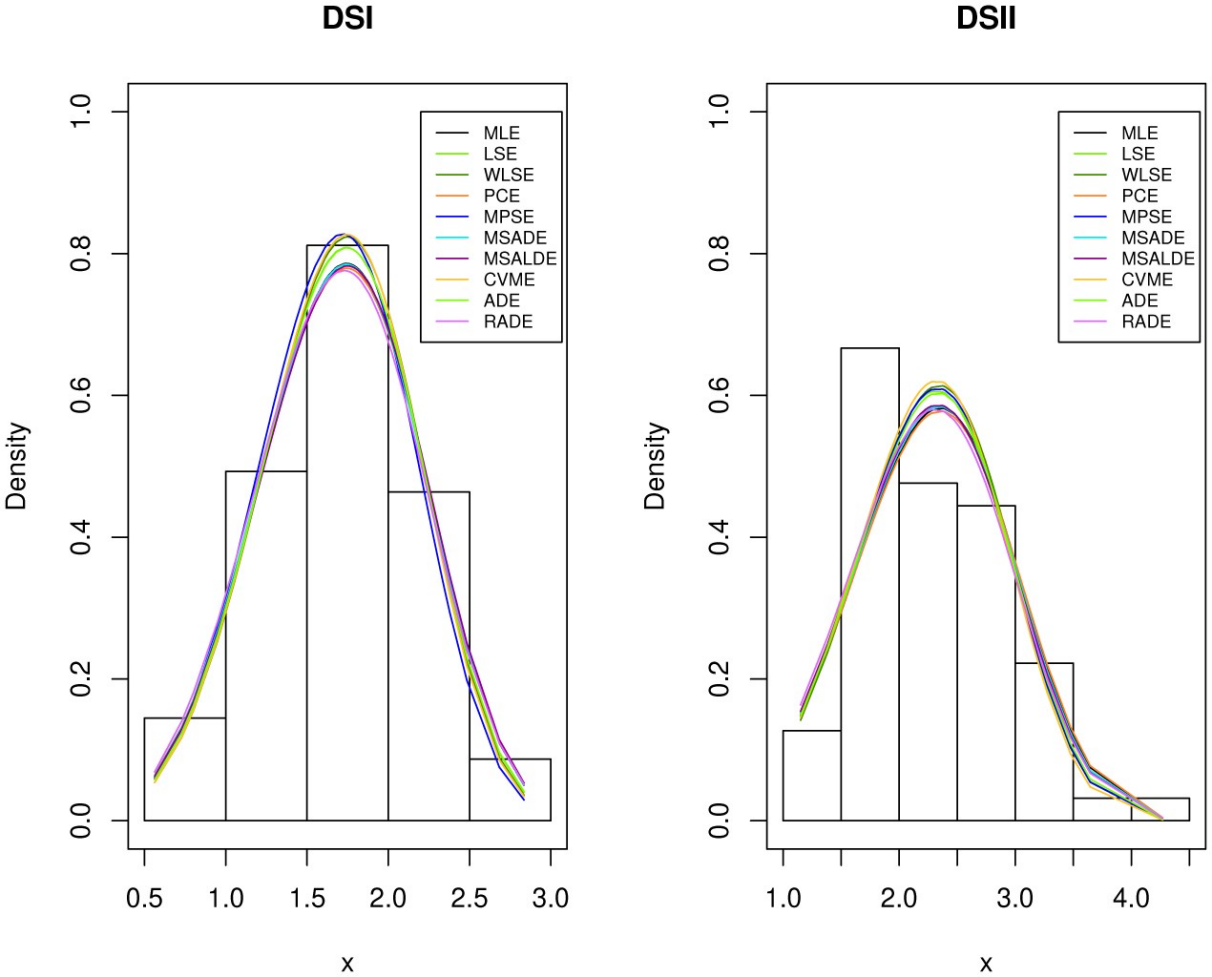
The histogram with the fitted PDFs for DSI and DSII.

From Tables 7 and 8, it is noted that the sample sizes are *m* = 69 and *r* = 63. Kundu and Gupta [3] analyzed these data after subtracting 0.75 from both the data sets. They showed that the Weibull distributions with the same shape parameter fit reasonably well to the transformed data sets. The fitted PDF, estimated CDF, estimated survival function and P-P plot of the Weibull distribution are displayed in Figs 1 and 2 for DSI and DSII, respectively. These Figure show that the Weibull distribution provides a suitable fit to these data. We use the transformed data to obtain the different estimates of *β*_1_, *β*_2_ and *α*, then we use these estimates to obtain the estimate of the stress-strength parameter using the different estimation methods. These values are displayed in Table 9. We also obtain the two bootstrap confidence intervals of the stress-strength parameter based on 1000 samples and display them in Table 10. We also computed the Kolmogorov-Smirnov(K-S) distance and the corresponding p-value based on the estimates MLEs, LSEs, WLSEs, PCEs, MPSEs, MSADEs, MSALDEs, CMEs, ADEs and RTADEs. These values are presented in Table 11. The results in Table 11 show that all considered methods provide satisfactory fits to the two data sets. The histograms of DSI and DSII and the fitted PDFs are displayed in Fig 3. These plots confirm the results in Table 11.

**Table 7:** Data set I.

1.312	1.314	1.479	1.552	1.700	1.803	1.861	1.865	1.944	1.958
1.966	1.997	2.006	2.021	2.027	2.055	2.063	2.098	2.140	2.179
2.224	2.240	2.253	2.270	2.272	2.274	2.301	2.301	2.359	2.382
2.382	2.426	2.434	2.435	2.478	2.490	2.511	2.514	2.535	2.554
2.566	2.570	2.586	2.629	2.633	2.642	2.648	2.684	2.697	2.726
2.770	2.773	2.800	2.809	2.818	2.821	2.848	2.880	2.954	3.012
3.067	3.084	3.090	3.096	3.128	3.233	3.433	3.585	3.585	

**Table 8:** Data set II.

1.901	2.132	2.203	2.228	2.257	2.350	2.361	2.396	2.397	2.445
2.454	2.474	2.518	2.522	2.525	2.532	2.575	2.614	2.616	2.618
2.624	2.659	2.675	2.738	2.740	2.856	2.917	2.928	2.937	2.937
2.977	2.996	3.030	3.125	3.139	3.145	3.220	3.223	3.235	3.243
3.264	3.272	3.294	3.332	3.346	3.377	3.408	3.435	3.493	3.501
3.537	3.554	3.562	3.628	3.852	3.871	3.886	3.971	4.024	4.027
4.225	4.395	5.020							

**Table 9:** Different estimates of *β*_1_, *β*_2_, *α* and *R* for real data.

	Method									
Parameter	MLEs	LSEs	WLSEs	PCEs	MPSEs	MSADEs	MSALDEs	CMEs	ADEs	RTADEs
*β*_1_	0.0862	0.0824	0.0780	0.0870	0.0854	0.0865	0.0852	0.0784	0.0818	0.0920
*β*_2_	0.0269	0.0263	0.0237	0.0275	0.0251	0.0276	0.0279	0.0244	0.0256	0.0305
*α*	3.8768	3.9769	4.0596	3.8497	4.0139	3.8712	3.8733	4.0670	3.9803	3.8030
*R*	0.7624	0.7576	0.7669	0.7597	0.7729	0.7580	0.7531	0.7622	0.7612	0.7509

**Table 10:** Different bootstrap confidence intervals of *R* for real data.

	Method	
Method	Boot-P	Boot-BCP
MLEs	(0.694,0.825)	(0.690,0.823)
LSEs	(0.668,0.826)	(0.674,0.833)
WLSEs	(0.691,0.833)	(0.692,0.831)
PCEs	(0.680,0.820)	(0.694,0.832)
MPSEs	(0.704,0.834)	(0.677,0.819)
MSADEs	(0.467,0.880)	(0.623,0.931)
MSALDEs	(0.665,0.825)	(0.676,0.834)
CMEs	(0.677,0.836)	(0.672,0.830)
ADEs	(0.682,0.826)	(0.684,0.826)
RTADEs	(0.683,0.828)	(0.658,0.811)

**Table 11:** The KS distance and p-value for different methods for DSI and DSII.

	Method									
Parameter	MLEs	LSEs	WLSEs	PCEs	MPSEs	MSADEs	MSALDEs	CMEs	ADEs	RTADEs
DSI										
K-S	0.0470	0.0377	0.0365	0.0498	0.0466	0.0464	0.0511	0.0388	0.0385	0.0493
p-value	0.9981	1.0000	1.0000	0.9955	0.9983	0.9980	0.9937	0.9999	1.0000	0.9961
DSII										
K-S	0.0768	0.0780	0.0780	0.0777	0.0742	0.0789	0.0801	0.0738	0.0753	0.0872
p-value	0.8510	0.8383	0.8381	0.8417	0.8783	0.8280	0.8137	0.8828	0.8669	0.7242

## Conclusion

In this paper, we compared ten estimation methods of stress-strength parameter *R* = *P*(*Y* < *X*), when *X* and *Y* are two independent Weibull distributions with equal shape parameter. The methods are: the maximum likelihood, least square, weighted least square, percentile, maximum product of spacing, minimum spacing absolute distance, minimum spacing absolute-log distance, method of Cramér-von Mises, Anderson-Darling and Right-tail Anderson-Darling estimators. We also considered two parametric bootstrap confidence intervals of *R*, namely, the percentile bootstrap and bias corrected percentile bootstrap confidence intervals. The performance of the differenr estimators was compared by conducting an extensive Mont Carlo simulation study and via analysing one real data set. In presence of small samples the method of maximum product of spacing is perform better than the maximum likelihood method. Overall, the simulation results show that the percentile and maximum product of spacing methods perform better than the other methods in terms of minimum relative mean square error and confidence interval length in most of the cases. The bias corrected percentile bootstrap confidence intervals perform better than percentile bootstrap confidence intervals in terms of minimum confidence interval lengths. We recommend to use the maximum product of spacing methods to estimate the stress-strength parameter of the Weibull distributions especially when the sample size is small. A future work is to estimate *R* = *P*(*Y* < *X*) using the different estimation methods when the shape parameters are not the same. Another future work is to study and compare the Bayesian estimation based on maximum likelihood and based on maximum product of spacing to estimate the stress-strength reliability of Weibull distribution.
